# MALDI-TOF mass spectrometry discriminates drug-susceptible and -resistant strains in *Mycobacterium abscessus*

**DOI:** 10.1371/journal.pone.0319809

**Published:** 2025-03-26

**Authors:** Tran Duong Thai, Nut Nithimongkolchai, Benjawan Kaewseekhao, Janejira Samarnjit, Chutipapa Sukkasem, Lumyai Wonglakorn, Auttawit Sirichoat, Arnone Nithichanon, Kiatichai Faksri

**Affiliations:** 1 Department of Microbiology, Faculty of Medicine, Khon Kaen University, Khon Kaen, Thailand; 2 Research and Diagnostic Center for Emerging Infectious Diseases (RCEID), Khon Kaen University, Khon Kaen, Thailand; 3 Clinical Laboratory Section, Srinagarind Hospital, Faculty of Medicine, Khon Kaen University, Khon Kaen, Thailand; Ramathibodi Hospital, Mahidol University, THAILAND

## Abstract

*Mycobacterium abscessus* (*M. abscessus*) infection is a significant public-health concern due to its resistance to multiple antibiotics and associated treatment challenges. There is a pressing need for a rapid and effective method capable of reliably identifying *M. abscessus* drug resistance. Our study aimed to investigate the capacity of matrix-assisted laser desorption/ionization–time-of-flight mass spectrometry (MALDI-TOF MS) to identify *M. abscessus* drug-resistant isolates, offering potential proteomic spectrum markers for detecting resistant strains in clinical diagnosis and treatment. With the aid of machine learning, particularly the decision-tree algorithm, predictive models demonstrated excellent performance with 100% sensitivity and specificity. Peaks at 4,062 Da, 7,518 Da, 8,359 Da and 2,493 Da were potential biomarkers that can distinguish between phenotypes resistant or susceptible to amikacin, linezolid, clarithromycin and cefoxitin, respectively. Besides diagnosing these phenotypes, the combination of machine learning and MALDI-TOF can identify patterns of resistance and susceptibility to various drugs in serially sampled isolates. In an analysis of nine serially collected samples from a single patient, MALDI-TOF could differentiate between *M. abscessus* strains resistant to three drugs—amikacin, linezolid and clarithromycin—and those completely susceptible to these drugs, based on distinct peak intensities. Furthermore, alterations in the patterns of amikacin and clarithromycin resistance/susceptibility influenced the MALDI-TOF spectra in serial isolates, whereas changes in susceptibility to linezolid did not affect the patterns. Hence, MALDI-TOF could be considered an efficient and dependable method for identifying *M. abscessus* drug resistance. This diagnostic tool has the potential to streamline the traditionally lengthy process of antimicrobial susceptibility testing while maintaining reliable results.

## Introduction

Nontuberculous mycobacteria (NTM) are opportunistic pathogens that cause symptoms similar to tuberculosis but require different treatment [1]. NTM organisms are life-threatening agents due to their intrinsic resistance to various antibiotics, making them difficult to treat and often leading to treatment failure [2]. *Mycobacterium abscessus* (*M. abscessus*) is a rapidly growing species of NTM. It is an emerging pathogen associated with a range of diseases including respiratory infections, disseminated disease and soft-tissue and bone infection [3,4]. This species is one of the most multidrug-resistant mycobacteria with cure rates often below 50% despite combination therapy with clarithromycin and amikacin for 6-12 months or more [5,6]. The prevalence of *M. abscessus* infections has significantly increased over the last 20 years [3]. In Northeast Thailand, *M. abscessus* is the most prevalent NTM pathogen [4] and treating it is a major problem for clinical microbiologists.

The Clinical and Laboratory Standards Institute (CLSI) recommends specific antimicrobials for testing susceptibility of *M. abscessus* to amikacin, cefoxitin, ciprofloxacin, clarithromycin, doxycycline (or minocycline), imipenem, linezolid, moxifloxacin, trimethoprim-sulfamethoxazole and tobramycin [7]. Amikacin remains a primary drug for *M. abscessus* treatment, with a low rate of *in-vitro* resistance [8]. Macrolides, particularly azithromycin and clarithromycin, are essential antibiotics in the therapeutic regimen for *M. abscessus* isolates with a non-functional *erm*(41) gene [3,8]. Broth microdilution is the standard recommended by CLSI for susceptibility testing of *M. abscessus* [7]. Although the result of minimum inhibitory concentration (MIC) is typically read after between three and five days, there are several scenarios where a longer time might be necessary. For instance, the final interpretation for clarithromycin can take at least 14 days to ensure detection of inducible macrolide resistance [7]. For patients receiving extended antibiotic treatment (such as those with cystic fibrosis), an incubation period of over five days is necessary to ensure adequate growth [7]. These examples illustrate that the conventional approach is time-consuming and may lead to ineffective treatment.

Matrix-assisted laser desorption/ionization–time-of-flight mass spectrometry (MALDI-TOF MS) is a rapid and reliable technology widely used for bacterial species identification [9,10]. This technique is also potentially used to detect antibiotic-resistant bacterial strains [11]. Several MALDI-TOF-based studies have explored its potential in investigating β-lactamase activity and biomarker-linked drug resistance in various bacterial taxa such as the family Enterobacteriaceae, Gram-positive cocci and Gram-negative bacteria [11].

The application of MALDI-TOF MS for identifying drug resistance in *M. abscessus* has not yet been demonstrated. Therefore, in this study, we aimed to investigate the capacity of MALDI-TOF MS, combined with machine learning, to suggest proteomic markers predicting drug resistance of *M. abscessus*. We highlighted the potential capacity of this combination of methods to identify strains resistant to single or multiple drugs.

## Materials and methods

### Study workflow

**Fig 1:**
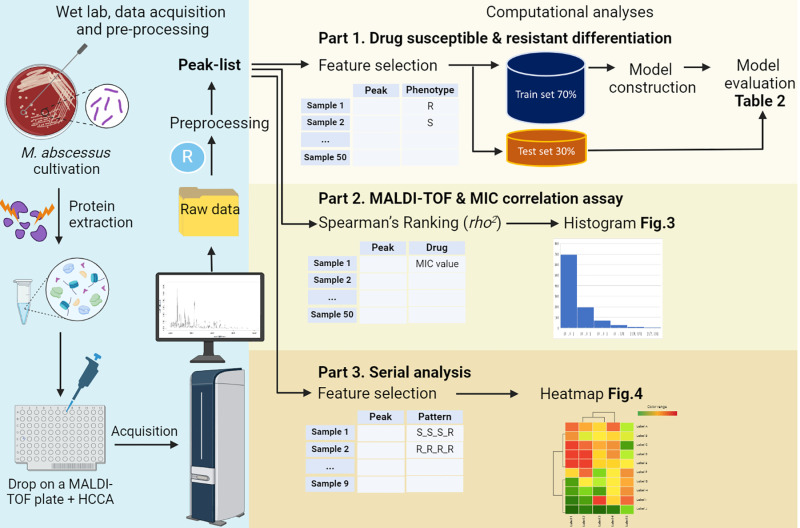
Schematic overview of experimental and analytic design.

This study workflow Fig 1 comprised two stages: Wet lab, data acquisition and processing and computational analyses. In the initial stage, fifty *M. abscessus* samples, were identified using whole-genome sequencing (WGS) [12], were cultured on blood agar for five days. Subsequently, one microliter of protein extracted from bacterial growth was placed on the MALDI-TOF plate with α-cyano-4-hydroxycinnamic acid (HCCA) for MALDI acquisition. The raw data retrieved from the mass spectrometer underwent preprocessing to generate a peak list using R programming MALDIquant R package [13].

In the computational analyses phase, the first part aimed to identify the significant peaks for differentiating drug-susceptible and -resistant phenotypes. Following a feature selection step using decision-tree algorithms, the dataset of important variable peaks was split into 70% for training and model construction, and 30% for testing and model evaluation.

The second part explored the correlation between peak intensity and MIC levels. This correlation was assessed using Spearman’s rank correlation and the correlation coefficient (*rho*^2^) values were represented in histograms.

The third part was to determine whether MALDI-TOF could distinguish between drug susceptibility and resistance in serial isolates of the same clone of *M. abscessus* obtained from a single patient. Spectra from nine serially collected samples underwent feature selection using decision-tree algorithms, and the distinct peak-expression patterns of drug-susceptible and -resistant isolates were displayed in a heatmap.

### Sample collection and drug susceptibility testing

Fifty *M. abscessus* samples collected in the period 2012 to 2018 from the biobank (S1 File), which had been maintained as stock cultures at the Clinical Microbiology Laboratory in Srinagarind Hospital, Khon Kaen University, Thailand, were recruited to this study. These isolates were identified using whole-genome sequencing (WGS) and retrieved from our previous study, along with their MIC levels [12]. Authorization to use bacterial samples was approved by the Khon Kaen University Ethics Committee for Human Research (HE591454). Research data were accessed between January 1^st^ 2024 and March 18^th^ 2024, with permission from the Khon Kaen University Ethics Committee for Human Research (HE661102). Since patient data was anonymized and de-identified prior to analysis, informed consent was not required for the use of medical data. The raw MALDI-TOF data from all bacterial samples in this study are freely available at Mendeley repository.

In our previous study [12], we tested drug susceptibility using the broth-microdilution method. Specifically, three to five colonies were selected from Löwenstein-Jensen (LJ) media using a loop and emulsified in demineralized water by vortexing with glass beads until a homogeneous suspension was achieved. If large clumps remained, only the supernatant was used. The suspension was then adjusted to a 0.5 McFarland standard. Fifty microliters of this suspension were transferred into a tube containing cation-adjusted Mueller–Hinton broth with TES buffer to achieve a concentration of 5 ×  10^5^ cfu/mL and mixed well. Subsequently, 100 µ L of this mixture was added to each well of a RAPMYCOL Sensititre 96-well plate (Sensititre, Trek Diagnostic Systems, United Kingdom), and the plate was sealed with an adhesive cover. The MIC plates were incubated at 37 °C in non-CO2 conditions and examined on day 5 [12].

We focused on four antibiotics (amikacin, linezolid, clarithromycin, and cefoxitin) in this study (S1 File). The MIC breakpoints of amikacin (AMK,) linezolid (LZD), clarithromycin (CLA) and cefoxitin (FOX) were 64 µg/mL, 32 µg/mL, 8 µg/mL and 128 µg/mL, respectively [7,14]. Based on these MIC breakpoints, the 50 isolates were divided into susceptible and resistant groups for each drug (Table 1). True infection and colonization were differentiated using published guidelines [4].

**Table 1:** Number of *M. abscessus* isolates and drug-susceptibility profiles.

Drugs	Minimum Inhibitory Concentration, MIC (µg/mL)													Drug susceptibility	
**≤0.06**	**0.12**	**0.25**	**0.5**	**1**	**2**	**4**	**8**	**16**	**>16**	**32**	**64**	**128**	**Resistant**	**Susceptible**
AMK^a^							8	28	7		4	3		3	47
LZD^b^							5	4	13		28			28	22
CLA^c^	1	2	6	4	3	2	5	7	15	5				27	23
FOX^d^											1	10	39	39	11

^a^Samples with MIC values ranging from 4 µg/ml to 32 µg/ml were placed in the “Susceptible group”, samples with MIC ≥  64 were placed in the “Resistant group”.

^b^Samples with MIC values ranging from ≤ 8 µg/ml to 16 µg/ml were placed in the “Susceptible group”, samples with MIC ≥ 32 were placed in the “Resistant group”.

^c^Samples with MIC values ranging from ≤ 2 µg/ml to 4 µg/ml were placed in the “Susceptible group”, samples with MIC ≥ 8 were placed in the “Resistant group”.

^d^Samples with MIC values ranging from ≤ 16 µg/ml to 64 µg/ml were placed in the “Susceptible group”, samples with MIC ≥ 128 were placed in the “Resistant group”

### Bacterial cultivation and protein extraction

*Mycobacterium abscessus* isolates were cultured on blood agar (Clinag Co., Limited, Thailand) for five days at 37 °C. A full loop (5-10 mg) of the culture was then transferred to a microcentrifuge tube containing 400 µ L of TE buffer. This tube was heated at 95 °C for 30 minutes and then frozen at -20 °C for one hour. After thawing at room temperature, the sample was centrifuged at 13,000 rpm for 2 minutes. The supernatant was discarded, and 900 µ L of ethanol was added. The sample was centrifuged again at 13,000 rpm for 2 minutes, and the supernatant was removed. The cells were washed with 900 µ L of sterile water and ethanol, each time followed by centrifugation at 13,000 rpm for 2 minutes. After the washing steps, the supernatant was discarded, and the sample was allowed to dry at room temperature. Glass beads of the same volume as the pellet, 20 µ L of formic acid and 20 µ L of acetonitrile were added. The sample was homogenized with an Ultrasonic processor VCX-750 (Sonics, Sonics & Materials, Inc.) for 5 minutes and then centrifuged at 13,000 rpm for 10 minutes. Before analysis, 1 µ L of the supernatant was placed on a MALDI target plate and allowed to dry at room temperature. Then, 1 µ L of HCCA (α-cyano-4-hydroxycinnamic acid) was added to the MALDI target plate and also allowed to dry at room temperature. The MALDI target plate was analyzed using an auto-flex max within an hour.

### MALDI-TOF acquisition

The MALDI-TOF acquisition was obtained using a mass spectrometer (Autoflex MALDI-TOF, Bruker Daltonics, Germany) with a 337 nm nitrogen laser. The machine was controlled using flexControl software (Bruker Daltonics, GmbH Bremen, Germany). The following settings were utilized: acceleration voltages for ion source 1 and ion source 2 were 25.00 kV and 23.45 kV, respectively, with lens voltages set to 6.0 kV. MALDI spectra of all isolates within a mass range of 2,000 to 20,000 Da were acquired, and these spectra were retrieved from the MALDI-TOF instrument using flexAnalysis software.

### MALDI-TOF analysis

The collected spectra underwent a filtering process to correct for measurement variations and systemic biases. This procedure was performed using conventional spectral data preprocessing techniques, which were modified from our previous study [10], with methodologies adapted from the MALDIquant R package [13]. The preprocessing consisted of intensity transformation (square root), intensity smoothing (Savicky-Golay with a half-window size of 20) [15], baseline correction (SNIP with 25 iterations), intensity normalization (total ion current), alignment (lowess algorithm), peak detection (MAD noise-estimation algorithm with an SNR of 2) and peak binning (tolerance of 0.002). The spectra results were gathered into a list of peaks, in which the columns indicated the positions of the mass and the rows showed the relative intensity of each isolate.

### Biomarker identification

The rpart R package [16] was used to identify important variable peaks based on discriminatory scores from all isolates that could distinguish between susceptible and resistant groups of each drug using a decision-tree algorithm. Then, the dataset was divided into training and test sets in a 70:30 ratio. In addition, the decision-tree algorithm from the same package was applied to construct a predictive model using important variable peaks and to detect biomarker peaks, with the parameter set as control =  rpart.control(minsplit =  1). The decision tree of the predictive model resulting from this was visualized using the rpart.plot R package [16]. A comparison of the relative intensity of biomarkers was statistically examined using the Kruskal-Wallis test for each drug.

Sensitivity, specificity and accuracy were computed from the test set as follows: sensitivity =  true positive/ (true positive +  false negative), specificity =  true negative/ (true negative +  false positive), positive predictive value (PPV) =  true positive/ (true positive +  false positive), negative predictive value (NPV) =  true negative/ (true negative +  false negative) and accuracy =  (true positive +  true negative)/ (true positive +  false positive +  true negative +  false negative). Sensitivity quantifies the model’s capability to provide a positive result when the isolate is resistant. Specificity measures the model’s ability to provide a negative result when the isolate is susceptible. PPV indicates the level of confidence we can have in the model when it predicts resistance, while NPV indicates the level of confidence we can have in the model when it predicts susceptibility.

### MIC values correlation and serial analysis through MALDI-TOF profiles

The correlation between MALDI-TOF peak intensity and MIC levels of all four drugs was examined using Spearman’s rank correlation coefficient in base R environment. Correlation coefficient (*rho*^*2*^) values computed from all mass peak and MIC levels, and their frequency were visualized using histograms.

Among the 50 isolates provided by our previous study, a series of nine *M. abscessus* isolates from a single patient were used to investigate patterns of drug susceptibility and resistance. These patterns were defined by the combination of drug-susceptible and -resistant phenotypes relating to four drugs: AMK, LZD, CLA and FOX. The important variable peaks able to distinguish the patterns were determined using a decision-tree algorithm in the rpart package [16]. The expression of relative intensity was plotted into a heatmap using the ComplexHeatmap R package [17].

## Results

### *Mycobacterium abscessus* susceptible and resistant phenotype groups differentiated by mass intensity of important peaks

**Fig 2:**
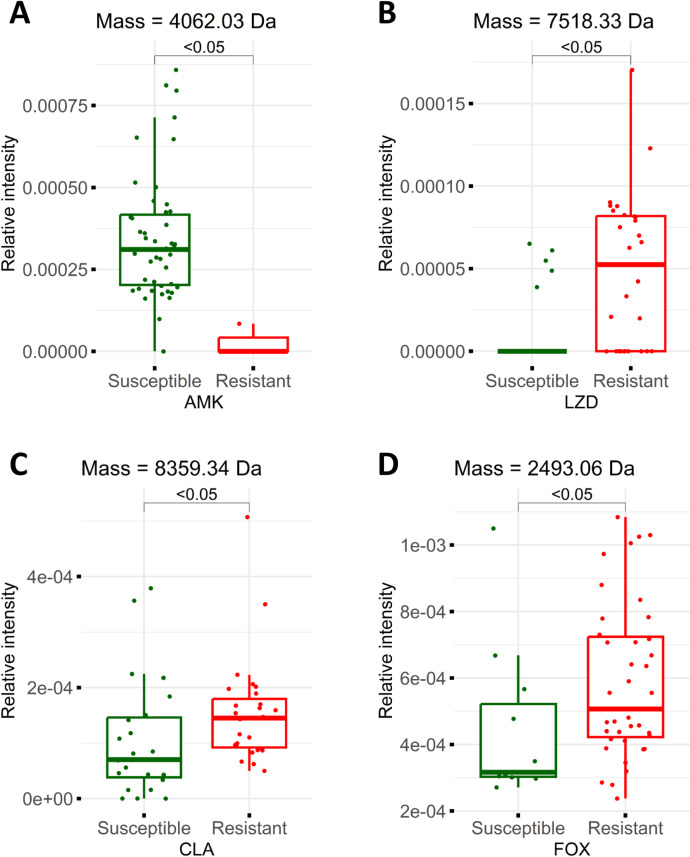
Boxplot of relative intensities of four candidate biomarkers among fifty samples: susceptible (green) and resistant (red) phenotypes for amikacin (AMK), linezolid (LZD), clarithromycin (CLA) and cefoxitin (FOX). The y-axis represents the relative intensity of the peaks and the x-axis indicates the two phenotype categories. The statistical difference in intensity among the two categories was calculated using the Kruskal-Wallis test. The phenotypes susceptible to AMK (A) demonstrated a higher relative intensity at peak 4,062 Da than the resistant phenotypes. (P-values =  0.005134). The phenotypes resistant to LZD (B), CLA (C) and FOX (D) at peaks 7,518 Da, 8,359 Da and 2,493 Da, respectively, had higher relative intensities than the susceptible phenotypes (P-value = 0.001124, P-value =  0.01379 and P-value = 0.03405, respectively).

After preprocessing, 1,008 mass peaks were listed from the 50 samples. To identify peaks useful for discriminating between drug-susceptible and -resistant phenotypes, all spectra were ranked based on discriminatory scores of relative intensities for each of the drugs—AMK, LZD, CLA and FOX—using the decision-tree algorithm. For phenotype classification of AMK, LZD, CLA and FOX, there were 7, 30, 29 and 29 peaks, respectively (S1 Fig). This was due to statistically significant differences in mass intensity, especially at 4,062 Da for AMK, 7,518 Da for LZD, 8,359 Da for CLA and 2,493 Da for FOX (Fig 2). The peak at 4,062 Da in phenotypes susceptible to AMK (Fig 2A) showed greater relative intensity compared to resistant phenotypes (with P-values <  0.05). Conversely, phenotypes resistant to LZD, CLA and FOX at peaks 8,359 Da (Fig 2B), 7,518 Da (Fig 2C) and 2,493 Da (Fig 2D), respectively, exhibited higher relative intensities than their susceptible phenotypes (with P-values <  0.05 in all cases). Therefore, the aforementioned peaks could potentially serve as biomarkers for *M. abscessus* drug-resistance identification.

The four predictive models developed from these important peaks, visualized in decision-tree figures (S2 Fig), demonstrated excellent performance, achieving 100% sensitivity, specificity, accuracy, PPV and NPV for predicting drug phenotype classification Table 2.

**Table 2:** Diagnostic performance of models for predicting resistant and susceptible phenotypes in our test set.

Drug susceptibility profile	AMK		LZD		CLA		FOX	
**Ref**	**Prediction**	**Ref**	**Prediction**	**Ref**	**Prediction**	**Ref**	**Prediction**
Resistant isolates	1	1	8	8	8	8	12	12
Susceptible isolates	14	14	7	7	7	7	3	3
Total	15 (100%)^*^		15 (100%)^*^		15 (100%)^*^		15 (100%)^*^	

* Sensitivity, specificity, accuracy, PPV and NPV

### MALDI-TOF mass peak intensity uncorrelated with MIC levels

**Fig 3:**
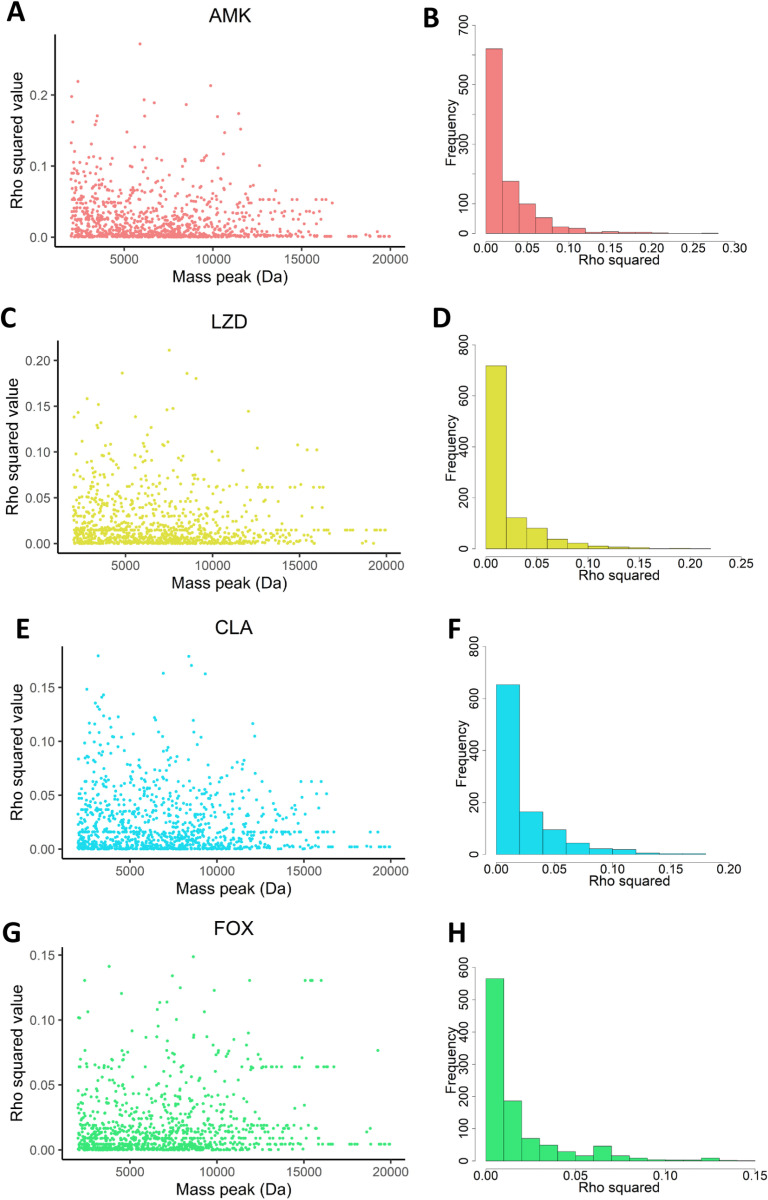
The distribution of correlation coefficient values between the intensity levels of 1,008 peaks and MIC levels for all four drugs, AMK (A), LZD (C), CLA (E) and FOX (G), using Spearman’s rank correlation coefficient. The frequency distribution of correlation coefficient values of 1,008 peaks and MIC levels for all four drugs, AMK (B), LZD (D), CLA (F) and FOX (H).

Initial results suggested that isolates with varying susceptibility and resistance showed differences in the intensities at certain mass peaks. Therefore, the second analysis was conducted to investigate further whether the peak intensity correlates with MIC levels. The correlation between the peak intensity of 1,008 distinct peaks and the MIC levels of four drugs, AMK, LZD, CLA and FOX, was examined using Spearman’s rank correlation coefficient (*rho*^*2*^). Correlation coefficient values close to 1 indicate a perfect association of ranks, whereas values close to 0 indicate no association. The distribution of Spearman’s rank coefficient for all four drugs were mostly between 0 and 0.1 (Fig 3A, 3C, 3E and 3G). In the total of 1,008 peaks, the number of peaks that had *rho*^*2*^ values nearly equal to 0 occupied the highest frequency in AMK (Fig 3B), LZD (Fig 3D), CLA (Fig 3F) and FOX (Fig 3H). Hence, our data suggested that no peak contained intensity correlated to the MIC levels, i.e., there was no correlation between mass peak intensity and MIC levels.

### MALDI-TOF can differentiate drug susceptibility and resistance by peak patterns in serially sampled clones

**Fig 4:**
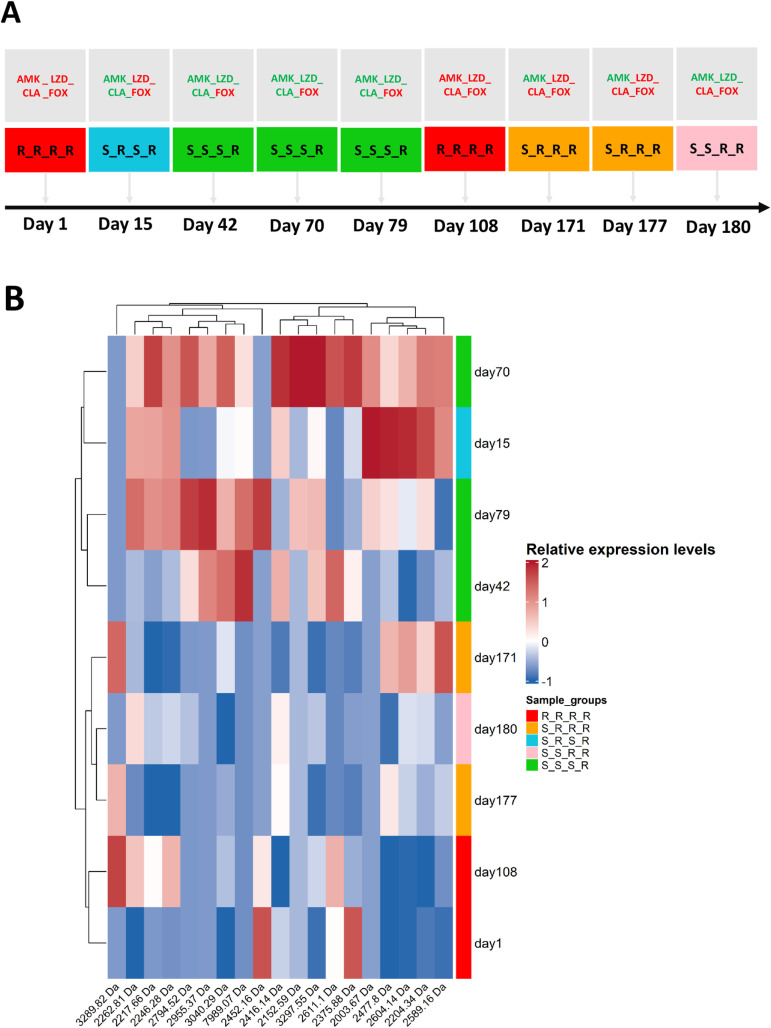
The sample drug patterns and relative expression levels of nineteen important peaks. Over the course of 180 days, nine serial samples were collected from a single patient. Each sample was determined as drug-susceptible (green) or -resistant (red) for AMK, LZD, CLA and FOX. The patterns of drug susceptibility and resistance are as follows: all resistant to AMK, LZD, CLA, FOX (R_R_R_R) (red), susceptible to AMK and CLA but resistant to LZD and FOX (S_R_S_R) (light blue), susceptible to AMK, LZD and CLA but resistant to FOX (S_S_S_R) (green), susceptible to only AMK (S_R_R_R) (orange) and susceptible to AMK and LZD with resistance to CLA and FOX (S_S_R_R) (pink) (A). Heatmap displaying patterns of drug susceptibility and resistance of 19 important variable peaks (B).

Analyzing for resistance/susceptibility for multiple drugs at the same time revealed that a pattern of peak values could inform about these. Treatment and monitoring of *M. abscessus* often involves serial sampling from one patient. Thus, we used a series of nine samples collected from one patient with persistent *M. abscessus* infection identified in our previous study (Kaewprasert et al., 2022) to determine whether MALDI-TOF could differentiate drug-susceptible and -resistant patterns in the same clone. In this series, samples were gathered over 180 days. Five distinct patterns were identified based on the phenotypes susceptible or resistant to four antibiotics. These patterns were arranged in the order AMK, LZD, CLA and FOX. The alterations in drug-resistance patterns were identified during the course of infection (**Fig 4A**). Nine spectra of serial isolates containing 1,008 peaks were ranked based on discriminatory scores of relative intensities using the decision-tree algorithm and provided clear 100% separation of each of five patterns (S3 Fig). Nineteen important variable peaks with differential intensity among the five patterns were then identified for the drug-susceptible and -resistant classification of AMK_LZD_CLA_FOX (S1E Fig). All nine isolates were FOX-resistant, so this category might not affect the classification process. According to the intensity levels of nineteen important peaks, the patterns fell into two clusters. In one cluster were samples showing complete resistance to AMK, LZD, CLA, FOX (R_R_R_R), susceptibility to only AMK (S_R_R_R) and susceptibility to AMK and LZD with resistance to CLA and FOX (S_S_R_R). The other cluster included samples with susceptibility to AMK, LZD and CLA (S_S_S_R) and resistance to LZD and FOX (S_R_S_R) (**Fig 4B**). In each cluster, the expression levels of peaks in a pattern did not change according to the time of sampling.

## Discussion

*Mycobacterium abscessus* infection presents a significant public health challenge due to its resistance to multiple antibiotics and the complexities associated with treatment. Therefore, there is an urgent need for a rapid and effective approach that can reliably detect *M. abscessus* drug resistance. While many studies have highlighted the potential of MALDI-TOF MS as a tool for identifying bacterial drug resistance [11], its application in understanding *M. abscessus* resistance remains unexplored. Thus, the scope of our study was to investigate the application of MALDI-TOF in identify drug resistance, which can further provide proteomic markers to detect resistant strains in clinical settings.

Using a combination of MALDI-TOF and machine learning, particularly decision-tree algorithms, we discovered that *M. abscessus-*resistant and -susceptible phenotypes can be differentiated due to notable differences in the intensity of certain mass peaks. These peaks are potential biomarkers for resistance against AMK (4,062 Da), LZD (7,518 Da), CLA (8,359 Da) and FOX (2,493 Da), antibiotics commonly used in the *M. abscesuss* treatment regimen. The predictive model based on these peaks that we developed from the decision-tree algorithm exhibited 100% sensitivity, specificity and accuracy. Using a similar approach, MALDI-TOF supported by machine learning was applied to screen *Campylobacter* spp. antimicrobial resistance [18]. Classifiers for ciprofloxacin and tetracycline, developed using random-forest algorithms to identify resistant strains of *Campylobacter spp*., demonstrated high accuracy, with sensitivities of 90.9% and 87.5%, respectively [18]. Several important peaks were identified for these classifiers, specifically 6436.22 Da, 2766.98 Da, and 2241.84 Da for the ciprofloxacin classifier, and 4365.25 Da, 2766.98 Da, and 7083.30 Da for the tetracycline classifier [18].

Similarly, MALDI-TOF is a rapid method to detect carbapenemase activity in Enterobacterales by screening for the presence or absence of the mass peaks at 300 and 489 Da, with 98.9% maximum sensitivity performance [19]. Expanding on this concept, in a large-scale study [20] involving more than 20,000 clinical samples, a machine-learning model was used to identify methicillin-resistant *Staphylococcus aureus* (MRSA) using MALDI-TOF MS. That study found that methicillin-resistant and -susceptible *S. aureus* could be discriminated based on the intensity differences of peaks in the range 6,590-6,999 Da. In particular, a peak at 6,593 Da indicated the presence of a crucial protein that could influence the binding affinity of the antibiotic in the MRSA group [20]. In addition to its use in bacterial studies, MALDI-TOF can also differentiate between azole-resistant and -susceptible strains of *Aspergillus fumigatus*. A specific peak at 4,359.5 Da was found in some azole-resistant strains, which have a 34-bp tandem repeat in the promoter region of cyp51A. Using random-forest and supervised linear support-vector algorithms, this method achieved a high accuracy of 80-97.06% in distinguishing these strains [21]. Although the models developed in the aforementioned studies might not have yielded 100% sensitivity, specificity and accuracy, values that we attained, their models were more realistic due to the larger sample size in training and test sets. Our study was limited to only fifty samples and there was an imbalance of isolates in susceptible and resistant groups, for instance, three versus forty-seven samples, respectively, for AMK diagnostic performance. The small number of included bacterial isolates can affect the discriminatory power and the realism of developing models, and the imbalanced dataset might cause bias and overfitting problems. Thus, more samples with an equal number of susceptible and resistant phenotypes of *M. abscessus* should be recruited in future studies.

In another approach, we utilized Spearman’s rank correlation coefficient to investigate whether lower or higher peak intensity was associated with the MIC levels for each drug. Consequently, those mass peaks could suggest proteomic markers that might be linked to drug resistance. The assay initially screened the correlation coefficient (*rho*^*2*^) values between intensity of 1,008 peaks from 50 samples and the MIC levels. We found no correlation between these two categories. This was probably because the MALDI-TOF data was derived from *M. abscessus* isolates which were grown without antibiotic treatment in various patients. Thus, several proteins that participated in the antibiotic-resistance process were not expressed and detected. In addition, the concentration of the prepared protein sample was not normalized before MALDI-TOF machine analysis. This could affect the comparative analysis among sample groups. Therefore, in further experiments, MALDI-TOF data of *M. abscessus* bacterial cells growing grown in the presence of each drug at that drug’s MIC level with protein concentration normalization should be included in future experiments.

Due to the long treatment required, it was possible to investigate (using MALDI-TOF MS) evolving changes in drug-resistance biomarkers in nine serial samples of a *M. abscessus* clone from a single patient. All nine isolates were resistant to FOX, implying that this category might not influence the classification procedure. Our results suggested that MALDI-TOF could differentiate between *M. abscessus* strains that were resistant to AMK+LZD+CLA and those entirely susceptible to these three drugs using the distinct peak intensities. In addition, the change of pattern of AMK and CLA can affect the MALDI-TOF spectra in serial isolates but this was not the case for LZD. Thus MALDI-TOF can identify varying patterns of antimicrobial resistance, including those involving multiple antibiotics simultaneously. This technique could therefore reduce time-consuming individual antibiotic susceptibility testing. However, definite biomarker candidates for each pattern have not yet been identified due to the limited number of serial isolates. A larger analysis of serial samples should be performed to investigate other antimicrobial patterns and develop models to diagnose drug phenotypic patterns of *M. abscessus.*

There was a lack of clinical data on patient responses throughout their course of treatment because this study used leftover bacteria from a prior study. This drawback emphasizes the need for real-time updates on patient reactions to treatment during serial sample collections in further studies. In serial sample analysis, the comparison of the MIC values and peak intensity was excluded because the nine serial samples limit the diversity of MIC values for each drug. To confirm a potential biomarker, we need correlations across a sufficiently wide range of MIC values. For example, among nine samples in our serial analysis, LZD has only two MIC values (16 and 32 µg/mL) (S1 File), which may lead to random correlations and unreliable outcomes. Ideally, serial sample recruitment should include a wide range of MIC values to effectively test the correlation using this method.

There were other limitations in this study. Changes in DNA and RNA expression, which likely reflect changes in *M. abscessus* drug resistance, were not investigated. Our study lacks data on DNA mutations, transcriptional regulatory factors, or RNA stability, all of which can influence the expression and synthesis of proteins related to drug resistance. Incorporating genomic and transcriptomic analyses could provide deeper insights into the mechanisms underlying drug resistance. This study was able to indicate the protein mass peaks that were important for drug resistance but could not specifically identify those proteins due to the lack of an appropriate database. The mass spectra obtained from the MALDI-TOF instrument were aligned with the bacterial identification range, which may have resulted in missing some proteins vital for recognizing drug resistance. Although antibiotics were selected from the 2011 CLSI guidelines, these are the only four drugs with MIC values that could be categorized clearly as ‘Susceptible’ or ‘Resistant. Because the bacterial isolates used were either entirely resistant or had only one susceptible value for other antibiotics, these antibiotics were unsuitable for developing machine-learning models. Thus, our study focused on AMK, LZD, CLA, and FOX. Despite the limitations outlined, our preliminary results offer practical guidance for both wet-lab and computational analyses which could lead towards an AMR detection tool based on MALDI-TOF.

## Conclusion

With the support of machine learning, we have demonstrated that MALDI-TOF serves as an effective and reliable method to identify drug resistance in *M. abscessus,* with the predictive models exhibiting excellent performance. Peaks at 4,062 Da, 7,518 Da, 8,359 Da and 2,493 Da are potential biomarker candidates to differentiate drug-resistant and -susceptible phenotypes of *M. abscessus* for AMK, LZD, CLA and FOX, respectively. In addition to diagnosing individual drug-susceptible and -resistant phenotypes, the combination of machine learning and MALDI-TOF can be used to detect patterns of resistance and susceptibility to various drugs in serially sampled isolates. Our diagnostic tools could potentially reduce the traditionally time-intensive process of antimicrobial drug-susceptibility testing while still delivering reliable results.

## Supporting information

S1 Fig Distribution of discriminatory score identified using decision-tree algorithms for four antibiotics; amikacin (A), linezolid (B), clarithromycin (C), and cefoxitin (D). Distribution of discriminatory score, identified using the decision-tree algorithm, of serial analysis among five drug patterns for the four aforementioned drugs using decision-tree algorithms (E).
(TIF)

S2 Fig Decision tree of predictive models developed using important variable peaks from decision-tree feature selection (Z-score scaled intensity). AMK (A), LZD (B), CLA (C) and FOX (D).
(TIF)

S3 Fig Decision tree of serial sample analysis. (TIF)

S1 File Sample information and MIC values. (XLSX)
