# Supplementary figures and images for: MALDI-TOF mass spectrometry discriminates drug-susceptible and -resistant strains in Mycobacterium abscessus
Source: PLoS One. 2025 Mar 26;20(3):e0319809. doi: 10.1371/journal.pone.0319809 (PMC11940421; doi:10.1371/journal.pone.0319809)

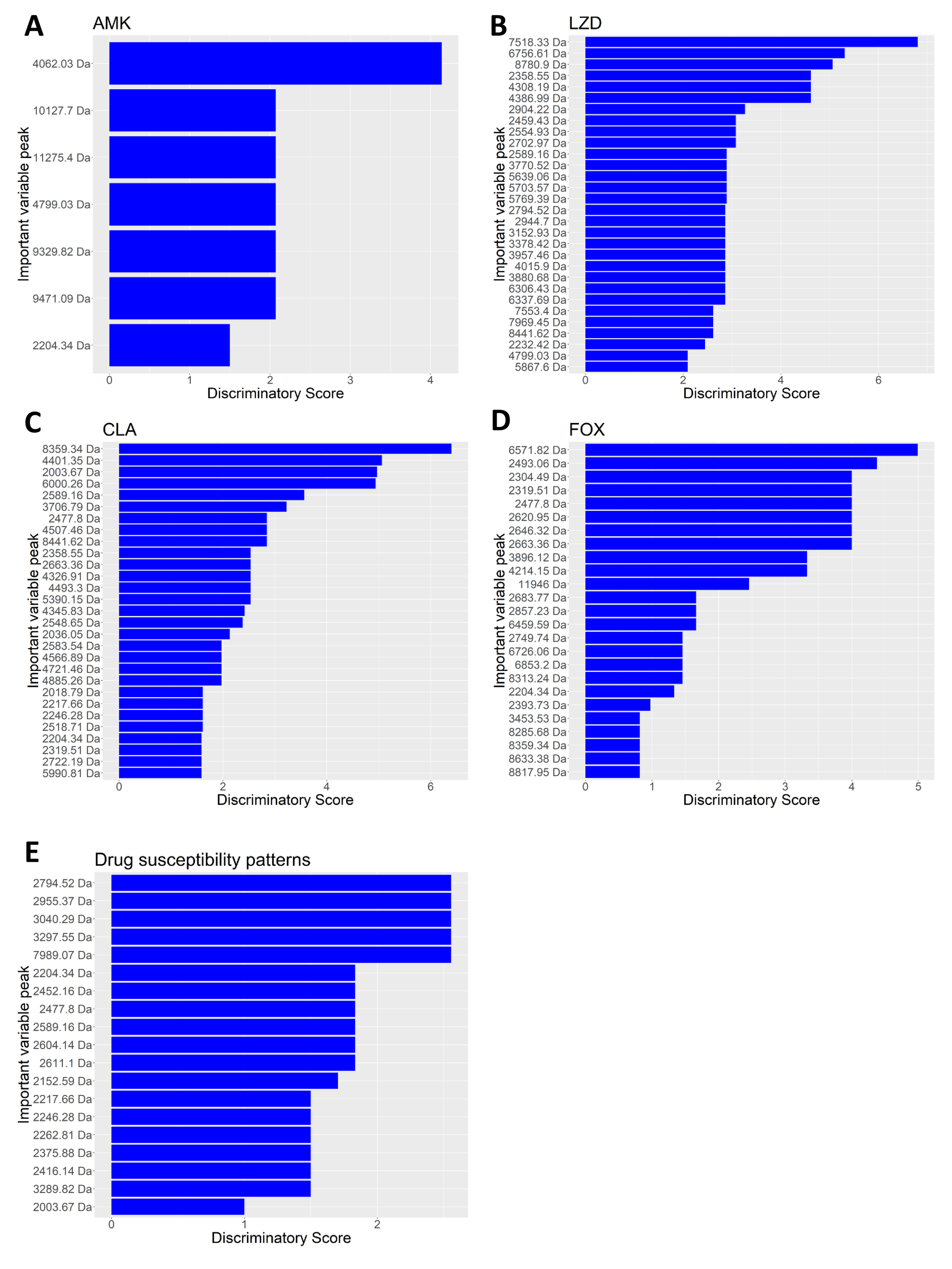

Supplement: S1 Fig — Distribution of discriminatory score, identified using the decision-tree algorithm, of serial analysis among five drug patterns for the four aforementioned drugs using decision-tree algorithms (E). (TIF) [file pone.0319809.s001.tif]

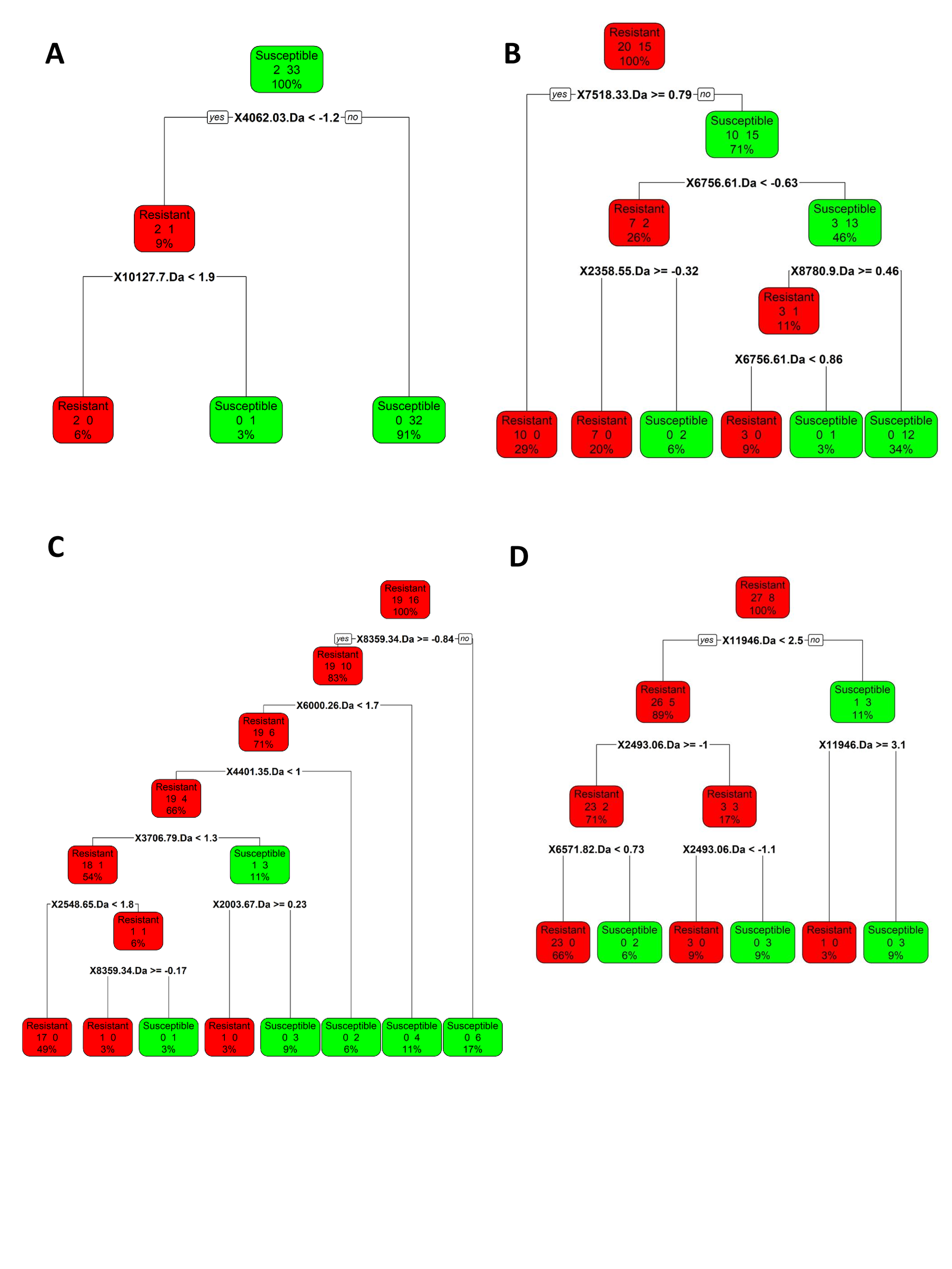

Supplement: S2 Fig — AMK (A), LZD (B), CLA (C) and FOX (D). (TIF) [file pone.0319809.s002.tif]

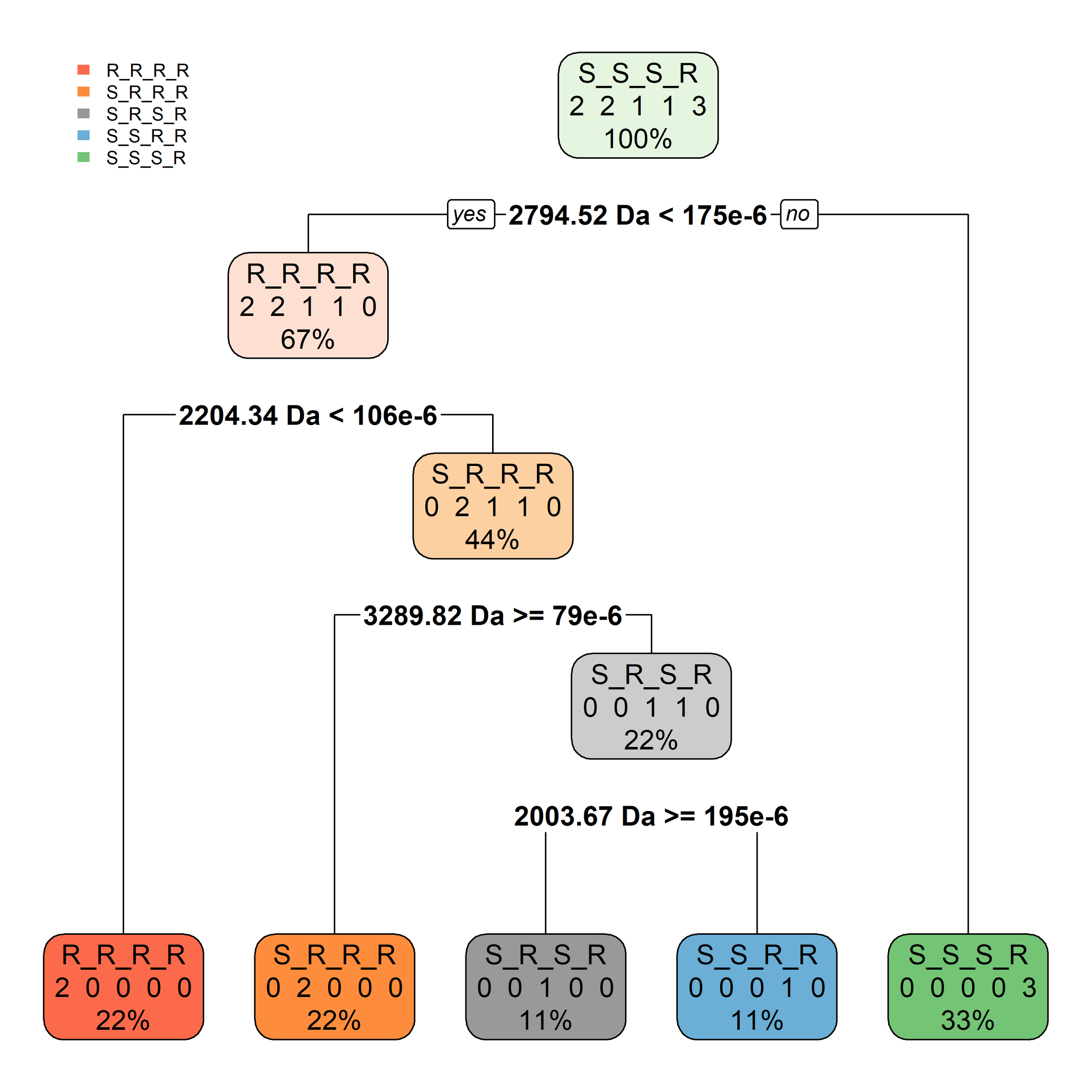

Supplement: S3 Fig — (TIF) [file pone.0319809.s003.tif]
